# Auditory Reserve and the Legacy of Auditory Experience

**DOI:** 10.3390/brainsci4040575

**Published:** 2014-11-14

**Authors:** Erika Skoe, Nina Kraus

**Affiliations:** 1 Department of Speech, Language, and Hearing Sciences, Department of Psychology Affiliate, Cognitive Science Program Affiliate, University of Connecticut, 850 Bolton Street, Storrs, CT 06105, USA; E-Mail: erika.skoe@uconn.edu; 2 Auditory Neuroscience Laboratory, Department of Communication Sciences, Institute for Neuroscience, Department of Neurobiology and Physiology, Department of Otolaryngology, Northwestern University, 2240 Campus Drive, Evanston, IL 60208, USA

**Keywords:** auditory system, neuroplasticity, sensitive periods

## Abstract

Musical training during childhood has been linked to more robust encoding of sound later in life. We take this as evidence for an auditory reserve: a mechanism by which individuals capitalize on earlier life experiences to promote auditory processing. We assert that early auditory experiences guide how the reserve develops and is maintained over the lifetime. Experiences that occur after childhood, or which are limited in nature, are theorized to affect the reserve, although their influence on sensory processing may be less long-lasting and may potentially fade over time if not repeated. This auditory reserve may help to explain individual differences in how individuals cope with auditory impoverishment or loss of sensorineural function.

## 1. Introduction

We borrow from the ideas of Brain Reserve and Cognitive Reserve in positing the existence of an Auditory Reserve. Reserve theory, which emerged from the study of dementia, seeks to explain why some individuals are more resilient to neuropathology than others [1,2,3]. This resilience, which is thought to emerge from the strengthening of existing neural networks and/or emergence of new networks, may change how a disease is expressed and potentially mask the symptoms of the disease, but not necessarily impede the progression of the disease. Cognitive Reserve and Brain Reserve are related but distinct concepts [3,4]: Brain Reserve Theory, also called Brain Maintenance, proposes that functional declines resulting from neuropathology can be explained by individual differences in neuroanatomy. Cognitive Reserve, in contrast, is specific to cognitive processes and posits that individual differences in lifestyle factors can account for individual differences in cognitive function that emerge due to neuropathology, with higher education, verbal ability, higher occupation status, social engagement, and knowledge of a second language each being associated with a stronger Cognitive Reserve [1,4,5]. The Cognitive Reserve is argued to build over time, beginning first in childhood [6], with the total reserve emerging from a lifetime of cognitive activity.

Translating these ideas into an Auditory Reserve, we postulate that auditory experiences early in life creates a neural scaffolding that affects auditory abilities later in life in ways that are either beneficial or detrimental depending on the nature of the earlier experiences. To help frame our hypothesis, in Section 2, we examine recent evidence about the persistence of short-term auditory experiences on automatic sound encoding within the human auditory system, after first reviewing evidence from animal models. Building on this body of evidence, in Section 3, we formulate our Auditory Reserve Hypothesis. In Section 4, we speculate on how the Auditory Reserve might emerge and evolve throughout an individual’s lifetime. The primary focus of this article is to introduce the Auditory Reserve Hypothesis and to make predictions for future research; however given the special focus of this issue of Brain Science on music, a secondary goal is to provide an overview of recent research on former musicians and place it within a larger theoretical framework.

## 2. Legacy of Early Experience: Evidence for the Auditory Reserve

### 2.1. Evidence from Animal Models

Although the capacity to undergo experience-dependent plasticity exists throughout an organism’s lifetime, it is typically greatest early in life. At both cortical and subcortical levels of the auditory system, early auditory experiences can have a profound and long-lasting effect on auditory development and, in turn, affect adult performance. This has been demonstrated across a series of studies over the last several decades involving animal models of auditory deprivation and chronic stimulus exposure [7,8,9,10,11,12]. For example, Oliver and colleagues [10] reported that rats exposed as neonates to an acoustically-impoverished auditory environment—consisting only of repeating, high-frequency tone pips—showed a residual of that early experience when tested later in life. The lasting effects of this chronic stimulation included increased auditory brainstem response (ABR) amplitudes and decreased ABR latencies to the frequency of overstimulation, in addition to expanded frequency maps within the inferior colliculus to the stimulation frequency [10]. However, as has been shown in gerbils and other species, auditory stimulation need not be impoverished for the effects to be long-lasting. Sarro and Sanes (2011) [13] reported that gerbils given brief auditory training as juveniles, showed superior performance over the control animals when retested as adults on the same tasks. A more recent study by the same group suggests that auditory training early in life may impart a lasting protective effect that can overcome early sensory deprivation (*i.e.*, conductive hearing loss) and promote auditory processing into adulthood [14]. These studies in animals set the stage for asking the question of whether the legacy of early experience on the auditory system can also be observed in humans.

### 2.2. Evidence from Humans: Musicians

Studies of life-long musicians have revealed that musical experience beginning in childhood facilitates the processing of music, as well as non-musical sounds, especially in conditions when the acoustical features of the sounds change rapidly over time or are less salient due background noise or reverberation [15,16,17,18,19,20,21]). One of the recurring themes within this line of research is that currently playing amateur musicians, regardless of age, have faster auditory system timing than untrained peers and less noisy responses [15,22,23,24,25]. In fact, fast neural timing is argued to be one of the hallmark features of the musician’s response to sound [24]. Recent longitudinal work is helping to confirm that neural differences observed between musician and non-musicians do not simply reflect inborn differences, but, instead, are the outcome of experience-dependent changes that arise over the course of musical training [26,27,28]. In addition, it has been demonstrated that middle-aged musicians who have been playing music since childhood have less age-related changes (*i.e.*, delays) in auditory system timing in addition to better auditory perception compared to non-musicians [21,29,30], suggesting that extended musical training might confer a protective effect that helps to slow-down biological aging. Such an interpretation is consistent with evidence that musical training allows for cognitive function to be stabilized with advancing age [31].

Two recent studies of former musicians from our group provide further evidence for the pivotal role of early auditory experience in setting the stage for later auditory abilities [32,33]. These two studies, which we review below, in conjunction with the animal literature summarized above, seeded the development of the *Auditory Reserve Hypothesis*.

#### 2.2.1. The Effects of Childhood Musical Training on the Mature Auditory System

**Figure 1:**
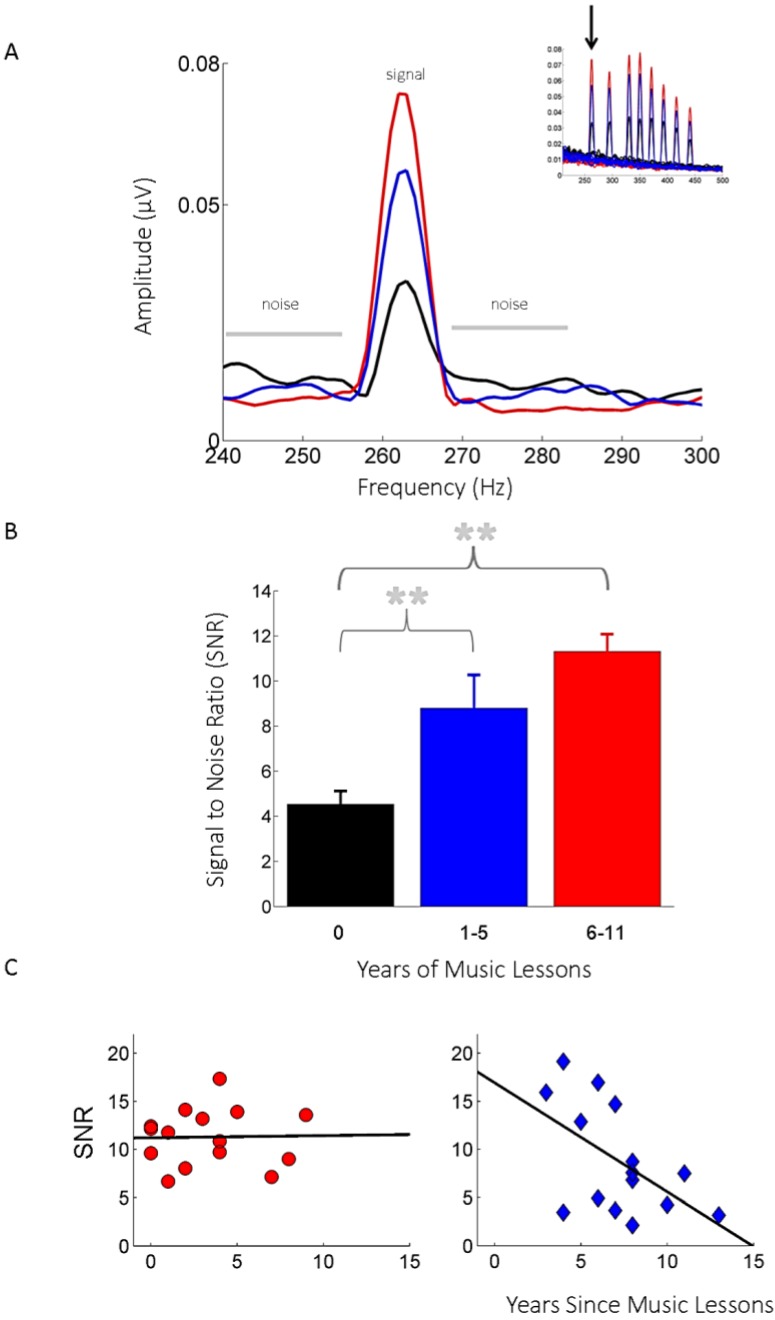
Young adults benefit from music practice during childhood. For adults with no past musical experience (**black**), the auditory brainstem frequency-following response is less robust (*i.e.*, smaller signal to noise ratios) relative to adults who started playing music around age 9 and who continued to play for either 1–5 (blue) or 6–11 (**red**) years. (**A**) Frequency-following responses (FFR) were recorded to 8 musical notes, varying in frequency from 262 Hz to 440 Hz (inset). The FFR was analyzed by calculating the amplitude of the response to each note relative to the physiological baseline (noise), producing a signal-to-noise ratio (SNR) for each note. (**B**) Bar graph depicting average SNR for each group (mean ± 1 SEM), ** *p* < 0.01. (**C**) Correlations between neural SNR and the number of years since music lessons occurred. In the group with 1–5 years of training (**right panel**), the SNR decreased in as a function of how long it has been since lessons occurred (*r* = −0.522, *p* = 0.046), whereas in the group with more training (**left panel**) this fading effect (*i.e.*, decrease) is not observed (*r* = 0.090, *p* = 0.747). Modified from Skoe and Kraus [32].

Skoe and Kraus (2012) examined auditory processing in young adults [18,19,20,21,22,23,24,25,26,27,28,29,30,31] who participated in music classes as children, either through group or private lessons [32] (Figure 1). On average, music lessons began around age nine, a typical time period for children to begin band or orchestra in school in the United States. We found that adults who played a musical instrument for one to five years as children had more robust neural responses to musical notes compared to age-matched untrained peers without any childhood musical training. This enhancement emerged as an increased signal-to-noise ratio (SNR), a measure reflecting the magnitude of the response relative to the background, neurophysiological noise. The neural enhancements observed in the group with one to five years of training matched what was seen in the group of individuals who began music lessons at the same age but continued to play a musical instrument for a longer period (a total of 6–11 years), suggesting initially that the effects of musical training where independent of the amount of training (see below). In the two musically trained groups, there was also an overall quieting of the response, as seen by a decrease in the physiological noise relative to the untrained group (F(2, 42) = 4.11, *p* = 0.023), which we speculate may enable more resilient auditory processing in acoustically-compromised conditions compared to the untrained group.

Overall, we interpret this pattern of results as evidence that young adults with past musical training continue to benefit from that past experience. However, an alternative interpretation is that the differences between the musically trained and untrained groups reflect pre-existing differences in auditory processing, with these individual differences underpinning which group began taking music lessons in the first place. While this alternative explanation may partially account for what makes the trained and untrained groups different, it cannot explain the differences observed between the two trained groups. In a follow-up analysis not reported in the original paper, we found that the SNR diminished as a function of how long ago the training occurred (*r* = −0.522, *p* = 0.046), suggesting that musical training had altered auditory processing but that the effect was not necessarily permanent and could diminish over time. Importantly, however, this correlation was observed only in the group with one to five years and not in the one with more training (*r* = 0.09, *p* = 0.747), raising the possibility that the effects of musical training become more long-lasting once a a critical threshold of training duration is met.

#### 2.2.2. The Effects of Childhood Musical Training on the Aging Auditory System

If the benefits of musical training extend after training stops, then how long do these neural benefits continue? The benefits of musical training appear to extend for multiple years [32], but what about decades? These were the questions recently addressed by White-Schwoch *et al.* 2013 [33] who examined the degree to which past musical training affects sensorineural timing in the aging auditory system. This study evaluated sensorineural timing in two groups of older adults (age range 55–76) who played musical instruments earlier in the life, and compared them to a third age-matched group with no musical training. The musically-trained participants were divided into groups based on whether they had a small amount of training (one to three years) or a more moderate amount (4–14 years). Across the two former musician groups, musical training occurred many decades prior to study enrollment (rnage: 37–59 years prior).

**Figure 2:**
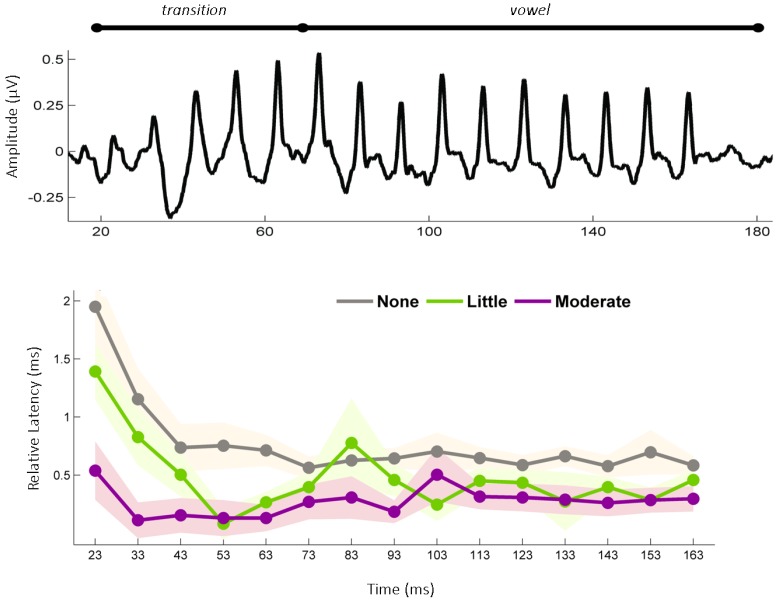
Older adults continue to benefit from music training that occurred early in life. (**Top**) Grand average auditory brainstem response from all participants to the speech sound (da). (**Bottom**) Participants were divided based on their childhood history of musical training into three groups. The “None” group (**gray**) represents those with no musical training, the “Little” group had between 1–3 years of musical training, and the “Moderate” group had between 4–14 years of musical training. The graph represents the relative latency of each prominent peak in the response to “da” presented in background noise, with the shaded regions representing 1 SEM. The larger the value on the ordinate, the greater the shift from the expected latency, and therefore the more delayed the response. Relative to the group with no training, the two groups with past musical experience have earlier latencies, during the first 50 ms corresponding to the “d” to “a” formant transition. Earlier neural timing is suggestive of a physiologically younger auditory system. Modified from White-Schwoch *et al.* [33].

The outcomes of the White-Schwoch *et al.* [33] study indicate that individuals who ceased playing a musical instrument decades ago continue to have a sensorineural advantage over untrained, age-matched peers. Specifically this study showed that older adults who were former musicians, had shorter subcortical response timing to sound compared to age-matched untrained controls (Figure 2). The effects were most apparent on the following conditions: (1) when the stimulus was presented in background noise, a type of auditory masker that can interfere with neural timing and impede speech perception; and (2) when the acoustic properties of the stimulus (*i.e.*, a “da” syllable) changed rapidly over time. These rapid acoustic changes, which occurred during the first 50 ms of the stimulus, help to impart phoneme identity (*i.e*., Is it a “da” or “ga”?), and temporal disruptions in encoding these acoustic changes are linked with impaired speech perception [22]. In this study, neural processing speed increased with the extent of past musical experience, suggesting that the duration of the experience is a critical factor that influences how much experience-dependent plasticity was initially expressed and then ultimately retrained. In addition to differing in the amount of musical training, the two former musician groups also differed on their self-reported proficiency on their instrument (more training was associated with higher proficiency ratings), which, in conjunction with recent evidence from bilinguals [34], may indicate that the end results of the initial experience, not just the duration of the experience, can affect the extent and persistence of neuroplasticity. However, because self-reported proficiency reflects an inherently subjective measure, future research should adopt more objective assessments of musical performance and second language proficiency to validate such claims.

## 3. Auditory Reserve Defined

As reviewed above, there is a growing body of research from human and laboratory animals indicating that auditory experiences during development can shape auditory processing later in life [10,13,32,33,35]. These studies led us to the idea of an Auditory Reserve, which we define as the ability to develop and maintain robust subcortical and cortical auditory functioning that enables an individual to preserve auditory processing abilities when faced with acoustically-challenging conditions and maintain robust sound-to-meaning connections when afflicted by disease or auditory deprivation. Depending on the strength of the reserve, auditory processing abilities, we speculate, may be partially if not fully preserved in such conditions. We further theorize that the *Auditory Reserve* is an emergent property of the auditory system, such that it reflects both the flexibility and durability of the networks comprising the auditory system.

By calling it a reserve, we are implying that the Auditory Reserve reflects neural resources that are accumulated over time and moreover that these resources may not be utilized only when needed. Like the Cognitive Reserve, we hypothesize that the Auditory Reserve is activity-dependent in the sense that it is affected by the level and nature of sensory stimulation that the individual has participated in throughout his or her lifetime. We propose that the Auditory Reserve is scaffolded by early auditory experiences and then strengthened, re-formed, or weakened by on-going experiences. The process of strengthening the reserve may result from the formation of additional neural networks that can be utilized during complex auditory tasks or when the auditory system is compromised by peripheral and central hearing loss.

## 4. Exploring the Auditory Reserve

In this section we explore some potential mechanisms by which an Auditory Reserve might emerge and flesh out a model that could be used for making predictions for future research.

### 4.1. Auditory Reserve: The Role of Auditory Enrichment and Impoverishment

Based on our current understanding of auditory system plasticity, we speculate that enriched auditory experiences fortify the reserve by strengthening and stabilizing existing networks, as well as creating additional networks for processing auditory information. We operationally define auditory enrichment as an increase in behaviorally-relevant interactions with sound in the environment or with self-produced sounds (e.g., speech, singing); by this definition, increased sound exposure would not constitute enrichment if the sounds were irrelevant background sounds or if the intensity of the exposure were potentially damaging to the auditory system.

We consider music to be just one type of enriching experience that could positively influence the reserve and suggest that other activities that increase the number of meaningful interactions with sound, such as mastering two languages or being an expert bird watcher (and learning many bird songs) [36], could possibly endow more resilient auditory circuitry across subcortical and cortical regions. In support of this, recent evidence suggests that musical training and bilingualism may strengthen auditory circuits and give rise to more resilient neural processing in acoustically-challenging conditions [16,19,24,37,38,39]. For example, Samelli and colleagues [25] have shown that, despite having an increased risk for sensorineural hearing loss, professional Pop/Rock musicians had earlier subcortical and cortical auditory responses than age-matched non-musicians, suggesting that musical training may mitigate the effects of hearing loss during adulthood. As further evidence, studies of older musicians indicate that sustained auditory enrichment minimizes the neural manifestations of hearing loss [30,40] and potentially slows the progression of age-related changes in auditory system function [21]. However, it is currently unknown whether musical training, or other forms of enrichment, might offer similar protective effects to children with hearing loss or who have experienced other forms of auditory deprivation (see [41]).

In contrast to enriched environments, we propose that impoverished auditory experiences may negatively contribute to the reserve by reducing neural synchrony and leading to fewer neurons engaged by sensory stimulation, with the outcome being weaker and less resilient auditory processing. Auditory impoverishment can take many forms including sensorineural hearing loss, repeated bouts of conductive hearing loss due to recurring Otitis Media (middle ear infection) [42], prolonged exposure to low-level noise [43], or reduced long-term exposure to structured or acoustically-diverse soundscapes [44]. We speculate that reduced access to stimulating environments on a chronic basis may undermine both the development and the retention of the Auditory Reserve and, in doing so, compromise communication between sensory and cognitive centers of the brain [44,45].

Future research should place an importance on studying the types and combination of experiences that strengthen or weaken the Auditory Reserve. Understanding what experiential factors influence the reserve could have major implications for designing remediation strategies that could slow or even prevent the progression of a disease, disorder, or natural aging or potentially even partially restore auditory system function in cases of sensorineural hearing loss.

### 4.2. Activation of the Auditory Reserve

We speculate that the facilitative or weakened nature of the reserve is most readily apparent when auditory processing is challenged by loss of sensory function (as a consequence of compromises to peripheral or central function), acoustic degradation (e.g., background noise, reverberation), or by acute changes in the environment (e.g., exposure to novel environments). In the case of musicians, sensory processing may be continuously bolstered by having a strengthened reserve, allowing current and former musicians to maintain enhanced sensory processing for various auditory tasks across the lifespan. Thus, musicians, as well as other groups who have undergone significant enrichment experiences (e.g., bilinguals), may always be actively drawing on the reserve, and this may explain why these groups outperform control groups on certain auditory tasks [16,18,19,24,37,38,39]. In addition, we speculate on the possibility that absolute pitch—the ability to identify pitch without a reference—may be an example of a kind of extraordinary reserve that reflects both genetic and environmental factors [46,47,48,49,50,51], given that the ability to process acoustically-degraded signals is aided by robust pitch processing [38,52].

We also leave open the possibility that facets of the Auditory Reserve may not manifest initially but may influence sensory processing only at specific ages or during auditory re-learning. This is, of course, not a new idea. In the human literature, there are numerous examples suggesting that older adults and younger adults utilize different neural networks to accomplish the same sensory, cognitive, and motor tasks and that the brain draws from compensatory networks to maintain function during the aging process or following stroke (e.g., [53,54,55,56]).

As a further precedent for the idea that the Auditory Reserve may not manifest until later in life, we point to evidence from animal models in the next section.

### 4.3. Activation of the Auditory Reserve: Evidence from Animal Models

In a landmark 2006 study, Kujawa and colleagues [23] showed that the effects of low-level exposure to noise in juvenile rats are not necessarily immediate, but instead that compromises to auditory function can emerge as the animal it ages. This study found that noise-exposed juveniles showed signs of accelerated age-related hearing loss including neural deterioration within the cochlea, suggesting that exposure to noisy environments during youth sets up a chain of events that manifest as widespread neural degeneration later in life. This findings lends credence to the idea that the effects of early life experience on the auditory system do not necessarily surface right away but can emerge later and, therefore, that the weakened status of the Auditory Reserve may not manifest until later in life, Additional evidence comes from Knudsen and colleague’s work on barn owls reared with prismatic spectacles [57,58]. Their studies indicate that earlier experiences impart a neurophysiological trace, which does not affect performance under normal environmental conditions, but does enable greater flexibility to respond to extreme changes in the environment. Knudsen *et al.* [57,58] found that adult owls who had experience wearing prismatic spectacles as juveniles were able to quickly adapt to their altered visual environment when refitted with spectacles as adult, as seen by rapid sound localization re-learning and corresponding shifts in interaural timing differences (ITD) within the central nucleus of the inferior colliculus, a central hub of the subcortical auditory system. Once the spectacles were removed, these adults were able to recalibrate to the “normal” auditory and visual associations. This ability to rapidly adapt to an earlier auditory environment, and fluidly transition between environments, may rely on anatomical connections in the adult brain that were formed as juveniles [58]. Important for our argument is that, although these earlier-formed connections remained into adulthood, they had no effect on localization abilities under normal conditions (*i.e.*, no spectacles). This suggests that there may be networks within the auditory system that may remain actively silenced or inhibited until needed.

### 4.4. Specificity of the Auditory Reserve: The Conditions that Allow an Earlier Experience to Affect Later Function

In barn owls, the effects of earlier localization experience are highly specific, in the sense that earlier localization experience affects later localization abilities without introducing meta-plasticity, an overall increase in all forms of neuroplasticity later in life [57]. This outcome in barn owls, however, is not necessarily surprising given the precise neural calculations needed for accurate localization, especially for a predatory species like the barn owl.

This specificity seen in barn owls, however, does not preclude the possibility that other types of experiences could have a more generalized effect later in life. Continuous musical training, for example, has been shown to lead to neural enhancements of musical stimuli [59,60], but also speech [59,61,62] and environmental sounds [63] (for reviews see [15,19,20]). This transfer of experience has been argued to be the outcome of overlapping neural networks for speech, music, and other complex sounds [20,64]. If the reserve helps to strengthen an aspect of music processing that is shared with speech, and/or if a network shared by speech and music is bolstered by the reserve, then this could provide a mechanism by which a musician or former musician may be able to capitalize on his/her reserve to facilitate how non-musical sounds are encoded. As part of the process of validating the concept of the Auditory Reserve, it will be crucial to understand which types of experiences have a specific and which have a more generic influence on the reserve, and, additionally, whether non-auditory experiences can influence the reserve.

### 4.5. The Auditory Reserve and Sensitive Periods

**Figure 3:**
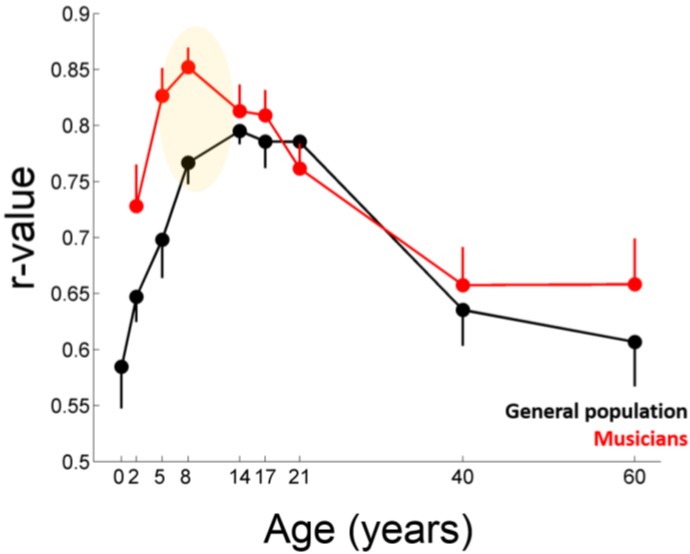
Developmental processes constrain how much the auditory environment can influence auditory function. As shown here for the auditory brainstem response to speech, the stability of the response (*i.e.*, within-test repeatability of the response) has a different developmental trajectory in musically trained individuals (**red**) compared to the general population (*i.e.*, individuals with no to minimal training) (**black**). Notably the group differences are largest early in life (before age 10) when the developmental trajectory is most in flux. Error bars represent one SEM. The value reported on the x-axis represents the youngest age for each group of participants. Modified from Skoe and Kraus, 2013 [74].

Sensitive periods are restricted windows during development when a particular experience can have a profound and lasting effect on specific areas of the brain and specific behaviors [65]. Given that experience-dependent plasticity is by definition greatest during sensitive periods, we postulate that the Auditory Reserve will be heavily influenced by experience occurring during these periods. Neural development is characterized by multiple, temporally overlapping sensitive windows, with different aspects of language learning (e.g., pronunciation, grammar, perception) and music skill acquisition (e.g., pitch perception, rhythm, motor learning) each having potentially different sensitive windows [65,66,67,68,69]. In addition, evidence from cochlear implanted children and animals suggests that the timeline for sensitive windows of auditory development may be different across subcortical and cortical auditory structures [70,71,72]. While each sensitive window may operate on its own timeline and have its own specific characteristics, we maintain the general claim that experiences that coincide with periods of developmental flux are expected to drive greater auditory system plasticity and, thereby, have a greater influence on the Auditory Reserve than experiences occurring when developmental processes are less active across the auditory system (Figure 3).

We have recently provided evidence that the developmental trajectory of the subcortical auditory system extends for a longer period than once believed and that sensitive periods for this component of the auditory system may consequently remain open until age 10, the point when development processes appeared to slow (Figure 3) [73,74,75]. Thus, in the case of the subcortical auditory system, this leads to the testable prediction that the reserve will be greatly informed by experiences occurring before age 10. We speculate that experiences occurring outside sensitive periods or which are limited in nature can influence the reserve but that their overall contribution may be diminished, such that their influence on sensory processing may be less long-lasting and may potentially fade over time if not reinforced (see Section 2.2).

While sensitive periods are typically expressed during early developmental time windows, recent work by Hensch’s and Merzenich’s groups is challenging the notion that sensitive periods can only occur early in life. For example, Hensch’s work is revealing that the molecular “brakes” that dampen experience-dependent plasticity during adulthood can be removed, allowing the timing of critical/sensitive periods to be pharmacologically controlled in laboratory animals [76] as well as humans [48]. Additionally, in animal models, there is evidence that enriched experience may lengthen (juvenile) sensitive periods [77], which together with the finding that extended exposure to noise can re-instate sensitive periods [78], raises the possibility that some forms of experience may be sufficient to re-instate sensitive periods in mature humans even without pharmacological manipulation. Related to this general idea, we have also recently speculated that the human auditory system may naturally undergo a second sensitive period later in life that begins when aging-related changes to auditory function emerge [74].

Other recent evidence in animal models suggests that sensitive periods are not limited to specific developmental windows but may, instead, be a characteristic feature of all neurons, even those generated during adulthood. Within the hippocampus, adult-born neurons exhibit an initial phase of plasticity followed by more stable properties, a pattern that resembles the postnatal plasticity seen in juvenile animals [79]. This cycle of “sensitive periods” whereby each generation of neurons undergoes experience-dependent plasticity followed by stabilization may allow young neurons, within a mature system, to be tuned by the current environment. Thus by continuously generating new neurons that have labile properties, this not only allows the organism to flexibly adapt to new environments throughout life, but it also provides a potential mechanism by which the nervous system can replenish the Auditory Reserve. However, whether similar phenomena occur in the auditory system is currently unknown and is a point of speculation.

There remain many open questions about the timing and instantiation of sensitive periods, including whether sensitive periods can re-open and whether there are side effects to this re-opening. In addition, there is the larger question of whether the results obtained from animal studies apply to the human brain given that humans have more complex communication capabilities than other animals. However, we speculate that regardless of when and how they occur, and what species is being considered, that the Auditory Reserve would be most directly influenced by the types of experiences that occurred within the most recent sensitive period.

### 4.6. Establishing the Auditory Reserve: The Role of *in Utero* Experiences

The human auditory system first responds to sound *in utero*, around the 22nd–24th gestational week [80,81], raising the possibility that experience-dependent changes can emerge during gestation. Neurophysiological evidence from cortical-evoked auditory responses suggest that the sounds heard *in utero* through the amniotic fluid influence how infants respond to sound, including music [82], after birth [83,84]. Whether the human subcortical auditory system undergoes experience-dependent plasticity *in utero* plasticity is unknown, maternal-related gestational factors—including stress, hormones, and narcotics—have been shown to affect how the central nervous system responds to sound, e.g., [85,86]). Recent evidence is further suggesting that such gestational factors can potentially alter the developmental timeline for speech perception [87]. As such, auditory experiences when the fetus is its earliest stages of hearing appear to affect how the sensory system develops and may, therefore, provide an initial foundation, or lack of foundation, for the establishment of the Auditory Reserve. If early auditory experiences are most informative, this sets up the rather provocative, yet speculative, idea that an abundance of auditory enrichment early in one’s life may offer a protective effect later in life and ward against the impact of later sensory impoverishment (e.g., hearing loss, environmental impoverishment) or auditory system pathology.

### 4.7. Interaction between the Auditory Reserve and Cognitive Reserve

In the final section of this paper, we come full circle by addressing the potential connection between the Auditory Reserve and Cognitive Reserve. Although we borrowed liberally from the notion of the Cognitive Reserve in putting forward the idea of the Auditory Reserve, there is reason to believe that they could share more than just a name and a few overlapping superficial characteristics.

There is diverse evidence to suggest that sensory and cognitive functions are coupled [24,34,37,45,88]. For example, Lin and colleagues have shown that hearing loss in older adults is associated with cognitive decline [88,89], although the underlying mechanisms of this association are unclear given that age-related changes in hearing loss often predate cognitive declines by many decades. As additional evidence, children with cochlear implants have been shown to be poorer at statistical learning in the visual domain despite normal visual acuity [90], suggesting that auditory experience is a gateway for developing certain domain-general cognitive processes [44,45]. It has also been shown in normal-hearing individuals that auditory function is tethered to complex cognitive processes underlying literacy, sustained auditory and visual attention, sentence processing in noise, and other higher-level tasks [15,37,91,92] (reviewed in [24]).

**Figure 4:**
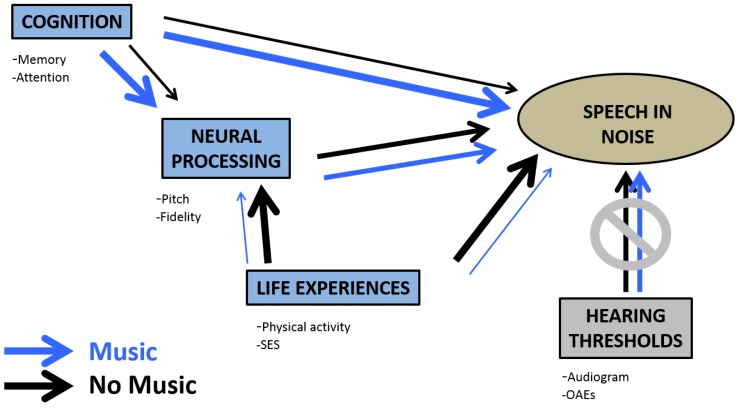
A model of processing speech in noise in middle-age and older adults. The structural equation model [93] includes variables relating to cognition, central auditory processing, musical background, and life experiences (*i.e.*, socioeconomic status and fitness level). This model, as schematized here, suggests that the neural networks that contribute to understanding speech in noise differ depending on whether the participant received musical training in their past or not. The group of older adults with past music training (**blue arrows**) was found to rely more on cognitive factors, whereas the group without musical training (**black arrows**) are shown to rely more on other life experiences. For each comparison, the wider arrow refers to the stronger contributor. Modified from Anderson *et al.*, 2013 [93].

In addition, recent modeling from our group [93] examined the interactions among peripheral hearing, central auditory processing, cognitive ability, musical experience, and other life experiences to predict how well middle-age to older adults could understand speech in a noisy background, a listening condition where the Auditory Reserve is expected to come into play. Building on evidence that neural networks involved in listening in noise are different between younger and older listeners [54], this study explored whether life experiences had an influence on the neural strategies that middle to older-aged adults use to understand speech in noise. Structural equation modeling suggested that adults with no past musical training draw on peripheral hearing and non-sensory factors such as intellectual and physical engagement to process speech in noise, whereas the same-aged adults with past musical training (even just a couple years of training) rely more on cognitive processes to aid their sensory perception in noise (Figure 4). This study reinforces the idea that a strong Cognitive Reserve could bolster the Auditory Reserve in aging individuals. However, in conjunction with data from bilinguals [5,34,94], it also speaks to the possibility that Auditory and Cognitive Reserves are not necessarily inherently coupled but that their coupling emerges through certain activities, such as playing music or speaking two languages.

## 5. Conclusions

In this article, we postulate the existence of an Auditory Reserve, a means by which auditory experiences and other life factors can aggregate to affect automatic sensory processing later in life. Examining the conditions that might support an Auditory Reserve, and the mechanisms needed to establish, maintain, and build this reserve, may provide insight into the natural mechanisms that the brain has in place to guard against sensory impoverishment and neural compromise. Understanding such mechanisms may then allow for their use as diagnostic and therapeutic tools. It should be noted that although our focus here is on the auditory system, the fundamental principles of our ideas may apply more generally to other sensory modalities. Future work will explore this possibility.
